# Far-Infrared Therapy Induces the Nuclear Translocation of PLZF Which Inhibits VEGF-Induced Proliferation in Human Umbilical Vein Endothelial Cells

**DOI:** 10.1371/journal.pone.0030674

**Published:** 2012-01-23

**Authors:** Yung-Ho Hsu, Yen-Cheng Chen, Tso-Hsiao Chen, Yuh-Mou Sue, Tzu-Hurng Cheng, Jia-Rung Chen, Cheng-Hsien Chen

**Affiliations:** 1 Department of Internal Medicine, Taipei Medical University-Shuang Ho Hospital, New Taipei City, Taiwan; 2 Department of Internal Medicine, Taipei Medical University-Wan Fang Hospital, Taipei, Taiwan; 3 Department of Biological Science and Technology, College of Life Sciences, China Medical University, Taichung, Taiwan; Istituto Dermopatico dell'Immacolata, Italy

## Abstract

Many studies suggest that far-infrared (FIR) therapy can reduce the frequency of some vascular-related diseases. The non-thermal effect of FIR was recently found to play a role in the long-term protective effect on vascular function, but its molecular mechanism is still unknown. In the present study, we evaluated the biological effect of FIR on vascular endothelial growth factor (VEGF)-induced proliferation in human umbilical vein endothelial cells (HUVECs). We found that FIR ranging 3∼10 µm significantly inhibited VEGF-induced proliferation in HUVECs. According to intensity and time course analyses, the inhibitory effect of FIR peaked at an effective intensity of 0.13 mW/cm^2^ at 30 min. On the other hand, a thermal effect did not inhibit VEGF-induced proliferation in HUVECs. FIR exposure also inhibited the VEGF-induced phosphorylation of extracellular signal-regulated kinases in HUVECs. FIR exposure further induced the phosphorylation of endothelial nitric oxide (NO) synthase (eNOS) and NO generation in VEGF-treated HUVECs. Both VEGF-induced NO and reactive oxygen species generation was involved in the inhibitory effect of FIR. Nitrotyrosine formation significantly increased in HUVECs treated with VEGF and FIR together. Inhibition of phosphoinositide 3-kinase (PI3K) by wortmannin abolished the FIR-induced phosphorylation of eNOS and Akt in HUVECs. FIR exposure upregulated the expression of PI3K p85 at the transcriptional level. We further found that FIR exposure induced the nuclear translocation of promyelocytic leukemia zinc finger protein (PLZF) in HUVECs. This induction was independent of a thermal effect. The small interfering RNA transfection of PLZF blocked FIR-increased PI3K levels and the inhibitory effect of FIR. These data suggest that FIR induces the nuclear translocation of PLZF which inhibits VEGF-induced proliferation in HUVECs.

## Introduction

Infrared radiation is invisible electromagnetic radiation, the wavelength of which is longer than that of visible light. According to differences in wavelength, the International Commission on Illumination (CIE) recommends dividing infrared radiation into the following three bands: near-infrared radiation (IR-A: 0.7∼1.4 µm), middle-infrared radiation (IR-B: 1.4∼3 µm), and far-infrared (FIR) radiation (IR-C: 3∼1000 µm). FIR therapy has the potential to improve endothelial function and reduce the frequency of some vascular-related diseases [1]–[5]. Recently, a clinical study evaluated the effect of FIR therapy on 145 hemodialysis (HD) patients with a native arteriovenous fistula (AVF), and found that FIR therapy improved inadequate access flow and survival of the AVF in HD patients through both thermal and non-thermal effects [6]. That study revealed that the non-thermal effects of FIR played a role in the long-term protective effect on vascular function.

The overwhelming disadvantage of an AVF in HD patients is its propensity for venous stenosis. A histological evaluation revealed that endothelial and fibromuscular hyperplasia is the leading cause of venous stenosis [7]–[9]. Intimal hyperplasia causes AVF intimal thickening with a large number of endothelial cells and a large amount of myofibroblast proliferation [9]. Recent studies in a variety of experimental arterial models of endothelial and smooth muscle injury suggested that macrophages, endothelial cells, and smooth muscle cells/myofibroblasts are all involved in the response to injury that is responsible for the development of neointimal hyperplasia [10], [11]. Potential growth factors and extracellular matrix proteins are thought to play roles in this process [12]–[15]. Vascular endothelial growth factor (VEGF) is particularly induced by hemodialysis graft placement, and then increases other mediators to cause the development of venous stenosis [16]. The influence of FIR on the function of VEGF is a critical problem in studying the biological activities of FIR on vascular function.

Many studies revealed that the biological activities of irradiation with FIR are highly associated with the endothelial nitric oxide (NO) synthetase (eNOS)/NO pathway. In rat models, the beneficial effects of FIR therapy on skin blood flow were suggested to be related to the L-arginine/NO pathway [5]. Akasaki et al. found that repeated FIR sauna therapy could induce angiogenesis by upregulating eNOS expression in mice with hindlimb ischemia [4]. Moreover, Ikeda et al. reported that 4 weeks of FIR sauna therapy significantly increased eNOS expression and NO production in cardiomyopathic hamsters with heart failure [2]. However, those studies did not investigate how FIR stimulates the eNOS/NO pathway. The molecular mechanisms of the biological activity of FIR irradiation need to be further elucidated.

In this study, we evaluated the biological effect of FIR on VEGF-induced proliferation in human umbilical vein endothelial cells (HUVECs). We found that a non-thermal effect of FIR induced translocation of the transcription factor, promyelocytic leukemia zinc finger (PLZF) protein, to nuclei, and ultimately inhibited VEGF-induced proliferation in HUVECs via the phosphoinositide 3-kinase/Akt signaling pathway.

## Materials and Methods

### Materials

VEGF and other chemicals of reagent grade were obtained from Sigma (St. Louis, MO, USA). The primary antibodies for PI3K p85, Akt, phospho-Akt (Ser 473), ERK1/2, phospho-ERK1/2 (Thr 202/Tyr 204), eNOS, and phospho-eNOS (Ser 1177) were purchased from Cell Signaling (Danvers, MA, USA). Primary antibodies for PLZF and GAPDH were respectively purchased from Calbiochem (Darmstadt, Germany) and AbFrontier (Seoul, Korea).

### Cell culture

The HUVEC line was purchased from the Bioresource Collection and Research Center (Hsinchu, Taiwan), and cultured in 90% medium 199 with 25 U/ml heparin and 30 µg/ml endothelial cell growth supplement (ECGS) adjusted to contain 1.5 g/L sodium bicarbonate with 10% fetal bovine serum. They were grown until the monolayer became confluent. The medium for culturing cells was then changed to serum-free medium, and cells were incubated overnight before the experiment. Cell culture reagents were obtained from Sigma-Aldrich (St. Louis, MO, USA).

### FIR exposure

A ceramic FIR generator, a WS TY301 FIR emitter (WS Far Infrared Medical Technology, Taipei, Taiwan), was used to provide FIR exposure. This FIR emitter generates electromagnetic waves with wavelengths in the range of 3∼25 µm. HUVECs were cultured in a 6-cm Petri dish with 3 ml medium for FIR exposure. During FIR exposure, an experimental group and a negative control covered with aluminum foil were set up in a culture chamber of a LiveCell™ system (Pathology Devices, Westminster, MD, USA) at 37°C with a 5% CO_2_ atmosphere. The FIR intensity was adjusted by changing the distance between the FIR emitter and culture chamber. The effective FIR intensity and wavelength received by HUVECs in culture dishes were detected and calculated by China National Infrared & Industrial Electrothermal Products Quality Supervision & Test Center (Wuhan, China). The effective primary FIR emission that the culture cells received ranged 3∼5 µm. Additionally, as a result of FIR exposure, there was secondary emission from the Petri dish lids with a peak wavelength of approximately 10 µm, calculated according to Wein's law.

### Cell proliferation assay

HUVECs (10^4^ cells/well) were cultured in a 96-well microtiter plate in a final volume of 100 µl/well of culture medium. After FIR exposure, cells were incubated at 37°C overnight. Cell proliferation was analyzed using a Quick Cell Proliferation Assay kit according to the instructions provided by the manufacturer (BioVision, Mountain View, CA, USA), and results are presented as the absorbance of each sample at 440 nm.

### Western blot analysis

In total, 15 µg of HUVEC lysate proteins was applied to each lane and analyzed by Western blotting. Peroxidase-conjugated anti-rabbit or anti-mouse immunoglobulin G (IgG) (at a 1∶5000 dilution) was used as the second antibody to detect primary antibodies by enhanced chemiluminescence (Thermo Scientific, Rockford, IL, USA). Data of protein bands on Western blots were also quantitated with QuantiScan software (Biosoft, Cambridge, UK).

### Measurement of nitrate/nitrite levels

Nitrate/nitrite in the culture medium was measured as described in a previous report [17]. In brief, a sample of medium was deproteinized with two volumes of 99% ethanol at 4°C, centrifuged (3,000 *g* for 10 min), and then injected into a collection chamber containing 5% VCl3. This strong reducing environment converted both nitrate and nitrite to nitric oxide (NO). A constant stream of helium gas carried the NO into an NO analyzer (Seivers 270B NOA; Seivers Instruments, Boulder, CO, USA), where the NO reacted with ozone, resulting in the emission of light. The light emitted was proportional to the quantity of NO formed; standard amounts of nitrate were used for calibration.

### Detection of intracellular reactive oxygen species (ROS)

Intracellular ROS production was detected with a fluorescent microscope using the fluorescent dye, 2′,7′-dichlorofluorescein diacetate (DCF-DA) (Molecular Probes, Eugene, OR, USA). Cells were cultured overnight on 4-well chamber slides (Nalge Nunc International, Naperville, IL, USA) at a concentration of 5×10^3^ cells per 400 µl culture medium. Before the experimental treatment, slides were preincubated with 10 µM DCF-DA at 37°C for 30 min in the dark, and then subjected to experiments. Fluorescence was observed by fluoromicroscopy with excitation and emission wavelengths of 485 and 530 nm, respectively.

### Nitrotyrosine formation assay

Nitrotyrosine formation was measured using Nitrotyrosine ELISA kits (Millipore, Billerica, MA, USA), following the manufacturer's instructions. A standard curve was used to determine the absolute concentration. Values were standardized to micrograms of protein for each sample.

### Phosphatase and tensin homolog (PTEN) activity assay

HUVECs were lysed at 4°C in lysis buffer (50 mM Tris, pH 7.5, 1% Nonidet P-40, 0.5% sodium deoxycholate, 150 mM NaCl, protease inhibitors). PTEN was collected by using immunoprecipitation kits (Roche Molecular Biochemicals, Mannheim, Germany) with specific antibodies and protein-G-agarose, following the manufacturer's instructions. Precipitates were washed with lysate buffer, and the activity of PTEN was analyzed by using PTEN Malachite Green Assay Kit (Upstate, Lake Placid, NY, USA). Absorbance was detected at 600 nm. Released phosphate was determined relative to a standard curve.

### RNA extraction and quantitative polymerase chain reaction (qPCR) analysis

Total RNA was extracted from HUVECs using the TRIzol method according to the protocol recommended by the manufacturer (Invitrogen, Carlsbad, CA, USA), and used to synthesize single-stranded complementary (c)DNA with a High-Capacity cDNA Reverse Transcription Kit (Applied Biosystems, Foster City, CA, USA). PI3K p85 subunit messenger (m)RNAs were quantified using TaqMan Gene Expression Master Mix (Applied Biosystems) with specific primers in an ABI 7300 Real-Time PCR System (Applied Biosystems). TaqMan Gene Expression Assay kits containing specific primers for PI3K p85-α (cat. no. Hs00933163_m1), PI3K p85-β (cat. no. Hs00178181_m1), and GAPDH (cat. no. Hs99999905_m1) were obtained from Applied Biosystems. Specific primers for GAPDH were used to normalize the amount of sample added. Relative amounts of PI3K p85 mRNA were quantitated using the comparative Ct method. All quantifications were performed on triplicate samples of three separate experiments.

### Nuclear protein purification

HUVECs were suspended in cold buffer A (containing 10 mM KCl, 0.1 mM EDTA, 1 mM DTT, and 1 mM PMSF) for 15 min, lysed by adding 10% NP-40, and then centrifuged at 5000×*g* to obtain nuclear pellets. The nuclear pellets were resuspended in cold buffer B (containing 20 mM HEPES, 1 mM EDTA, 1 mM DTT, 1 mM PMSF, and 0.4 mM NaCl), vigorously agitated, and then centrifuged.

### Short interfering (si)RNA transfection

PLZF siRNA (cat. no. sc-37149) and control siRNA (cat. no. sc-44234) were purchased from Santa Cruz Biotechnology. Cells were grown to 70% confluence, and PLZF siRNA and control siRNA were transfected using the TurboFect reagent according to the manufacturer's instructions (Fermentas, Glen Burnie, MD, USA). The final concentration of PLZF siRNA for transfection was 100 nM.

### Statistical analysis

A Student's *t*-test was used in all statistical tests. Distributions of continuous variables in groups were expressed as the mean±S.D. A value of *p* of <0.05 was considered to indicate statistical significance.

## Results

### FIR inhibits VEGF-induced proliferation in HUVECs

**Figure 1:**
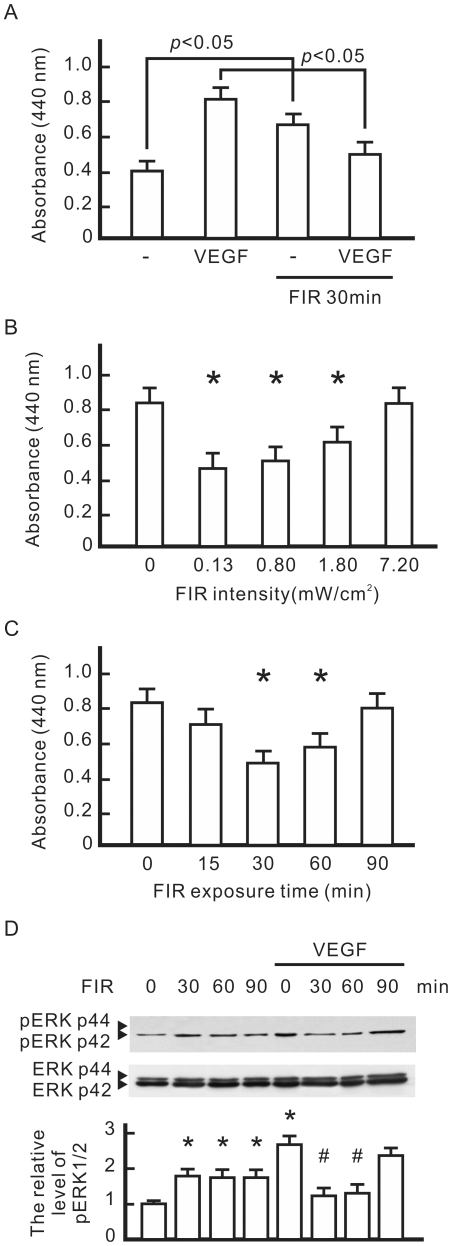
The influence of far infrared (FIR) exposure on VEGF-induced proliferation in HUVECs. (A) The influence of VEGF and FIR on cell proliferation. HUVECs were pretreated with VEGF (10 ng/ml) with or without FIR exposure at 0.13 mW/cm^2^ for 30 min, and then cultured overnight. Cell proliferation results are presented as the absorbance of each sample at 440 nm. (B) The influence of FIR intensity on VEGF-induced cell proliferation. VEGF-pretreated cells were exposed to FIR at the indicated intensity for 30 min, and then cultured overnight. *p<0.05 vs. the control without FIR exposure. (C) The influence of FIR exposure time on VEGF-induced cell proliferation. VEGF-pretreated cells were exposed to FIR at 0.13 mW/cm^2^ for the indicated periods, and then cultured overnight. *p<0.05 vs. the control without FIR exposure. (D) Western blotting of VEGF-induced phosphorylation of ERK1/2. HUVECs were pretreated with or without VEGF (10 ng/ml) for 30 min, exposed to FIR at 0.13 mW/cm^2^ for the indicated periods, and then cultured for 8 h. ERK1/2 was detected as a loading control. By comparison with the control without FIR exposure, the relative levels of phospho-ERK1/2 were obtained and are shown as the mean±S.D. from six determinations in three cell preparations. *p<0.05 vs. the control without FIR exposure. ^#^ *p*<0.05 vs. the VEGF-treated group without FIR exposure.

To evaluate the biological effects of FIR, VEGF-pretreated HUVECs were exposed to 0.13 mW/cm^2^ of FIR for 30 min and then cultured overnight. A cell proliferation analysis revealed that both VEGF and FIR exposure significantly increased cell proliferation in HUVECs (Fig. 1A). However, FIR exposure significantly inhibited VEGF-induced proliferation in HUVECs. In an intensity course analysis, effective FIR intensities of 0.13, 0.8, and 1.80 mW/cm^2^ significantly reduced VEGF-induced proliferation (Fig. 1B). This inhibitory effect of FIR peaked at the effective intensity of 0.13 mW/cm^2^ and decreased at higher FIR intensities. When the effective intensity rose to 7.20 mW/cm^2^, FIR failed to reduce VEGF-induced proliferation in HUVECs. Therefore, we applied 0.13 mW/cm^2^ as the working intensity in subsequent experiments. In a time course analysis, the inhibitory effect of FIR peaked at 30 min and decreased with longer exposure times (Fig. 1C). A western blot analysis was also used to evaluate the influence of FIR exposure on VEGF-activated extracellular signal-regulated kinase (ERK)1/2. As shown in Fig. 1D, FIR exposure significantly inhibited the phosphorylation of ERK1/2. Similar to the inhibitory effect of FIR on cell proliferation, the inhibitory effect of FIR on the phosphorylation of ERK1/2 peaked at 30 min of FIR exposure, and gradually decreased at longer exposure times.

### The inhibitory effect of FIR on VEGF-induced proliferation is associated with eNOS-mediated NO generation

**Figure 2:**
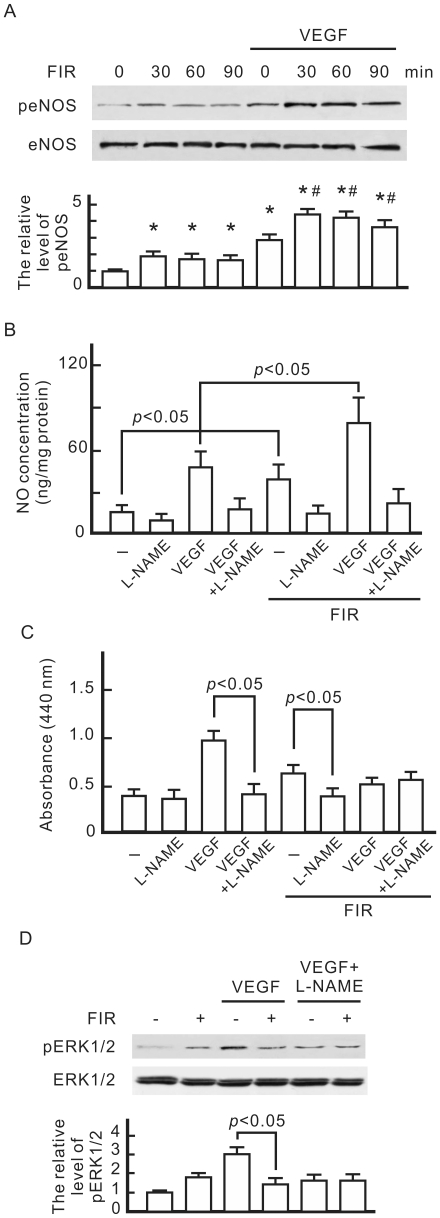
Involvement of eNOS and NO in the biological effect of FIR in HUVECs. (A) Western blotting of phospho-eNOS. HUVECs were pretreated with or without VEGF (10 ng/ml), exposed to FIR at 0.13 mW/cm^2^ for the indicated periods, and then cultured for 1 h. We detected eNOS as a loading control, and quantified phospho-eNOS expression relative to eNOS. By comparison with the control without FIR exposure, relative levels of phospho-eNOS were obtained and are shown as the mean±S.D. from six determinations in three cell preparations. *p<0.05 vs. the control with FIR exposure for 0 min. ^#^ *p*<0.05 vs. the VEGF-treated group with FIR exposure for 0 min. (B) Detection of FIR-induced NO. HUVECs were pretreated with VEGF and NG-nitro-L-arginine methyl ester (L-NAME) (5 mM) as indicated, exposed to FIR for 30 min, and then cultured for 1 h. The NO concentration in cultured medium was detected by a nitric oxide analyzer. (C) The influence of L-NAME on cell proliferation in HUVECs. HUVECs treated as indicated were cultured overnight. Data are shown as the mean±S.D. from six experiments. (D) Western blotting of phospho-ERK1/2. HUVECs treated as indicated were cultured for 8 h. ERK1/2 was detected as a loading control. By comparison with the control without FIR exposure, relative levels of phospho-ERK1/2 were obtained and are shown as the mean±S.D. from six determinations in three cell preparations.

A western blot analysis revealed that both VEGF and FIR exposure induced the phosphorylation of eNOS in HUVECs (Fig. 2A). FIR exposure further increased the phosphorylation of eNOS in VEGF-treated HUVECs. This induction peaked at 30 min and gradually decreased at longer exposure times. When we monitored the concentration of NO in the culture medium, FIR exposure further induced NO generation by HUVECs although VEGF also induced NO generation (Fig. 2B). These increases in NO generation were inhibited by NG-nitro-L-arginine methyl ester (L-NAME), an inhibitor of NOS. In cell proliferation tests, FIR exposure slightly increased the cell proliferation of HUVECs without VEGF treatment, but mitigated VEGF-induced proliferation (Fig. 2C). In L-NAME-treated HUVECs, VEGF-induced proliferation significantly decreased, and there was no further decrease due to FIR exposure. L-NAME also reduced VEGF-induced phospho-ERK1/2, which was not influenced by FIR exposure (Fig. 2D).

### The influence of a thermal effect on VEGF-induced proliferation differed from that of FIR

**Figure 3:**
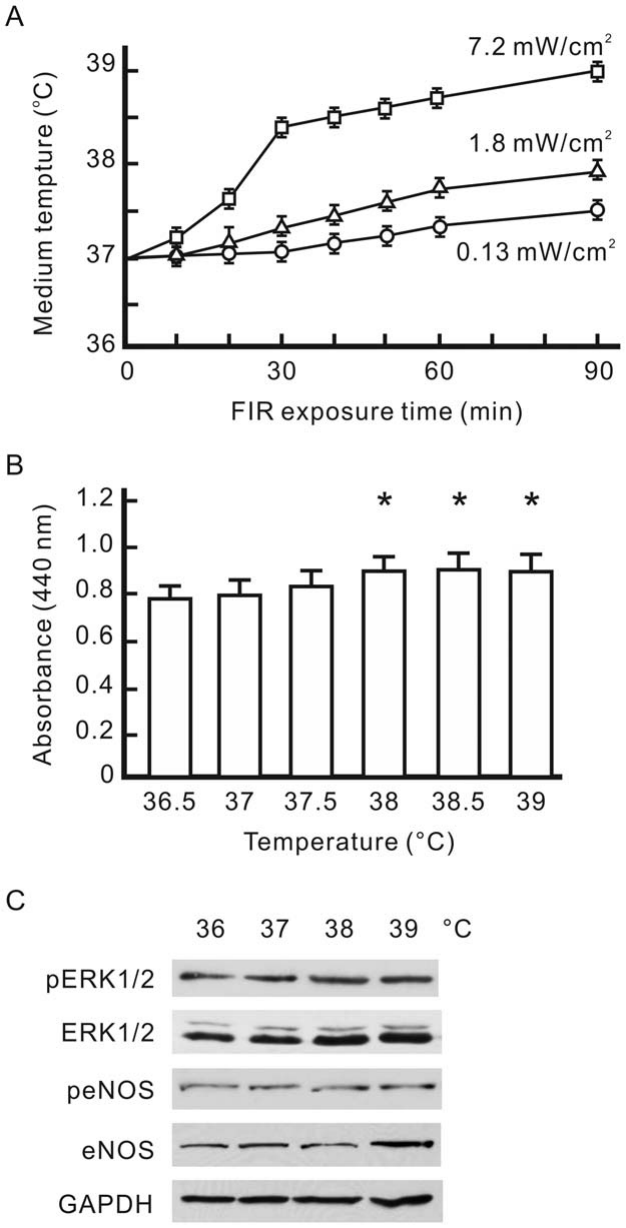
Influence of a thermal effect on VEGF-induced proliferation in HUVECs. (A) FIR exposure-increased the temperature of the culture medium. The temperature of 3 ml of culture medium in 6-cm culture dishes was detected after FIR exposure at 0.13, 1.8, or 7.2 mW/cm^2^ for the indicated periods. (B) The influence of thermal pretreatment on VEGF-induced cell proliferation. HUVECs were pretreated with VEGF (10 ng/ml), cultured at the indicated temperature for 30 min, and then cultured at 37°C overnight. Data are shown as the mean±S.D. from six experiments. * *p*<0.05 vs. the group at 37°C. (C) Western blotting of phospho-ERK1/2, ERK1/2, phospho-endothelial nitric oxide synthase (eNOS), and eNOS. HUVECs were pretreated with VEGF, cultured at the indicated temperature for 30 min, and then cultured at 37°C for 8 h. GAPDH was detected as a loading control.

A thermal effect of FIR on the cell culture system was also examined in this study. With FIR exposure at different effective intensities, we detected the temperature of 3 ml of culture medium in a 6-cm plate at different time points. The effective FIR intensity of 0.13 mW/cm^2^ only mildly heated the culture system, and raised the temperature of the medium from 37.0±0.1 to 37.1±0.1°C in 30 min (Fig. 3A). The higher effective FIR intensity of 7.2 mW/cm^2^ had a strong heating effect, which raised the temperature of the medium from 37.0±0.1 to 38.4±0.1°C in 30 min. The temperature of the medium rose along with the FIR intensity and exposure time. To evaluate the thermal effect on cell proliferation, we cultured VEGF-pretreated HUVECs at different temperatures for 30 min without FIR exposure, and detected VEGF-induced cell proliferation after overnight culture. As shown in Fig. 3B, pretreatment at 38∼39°C slightly increased VEGF-induced proliferation in HUVECs. A western blot analysis revealed that pretreatment at 38∼39°C did not influence VEGF-induced expression of phospho-ERK1/2 or phospho-eNOS although 39°C pretreatment induced eNOS expression (Fig. 3C). These expression patterns of phospho-ERK1/2 and phospho-eNOS differed from FIR-induced events.

### The inhibitory effect of FIR on VEGF-induced proliferation involved VEGF-induced ROS generation

**Figure 4:**
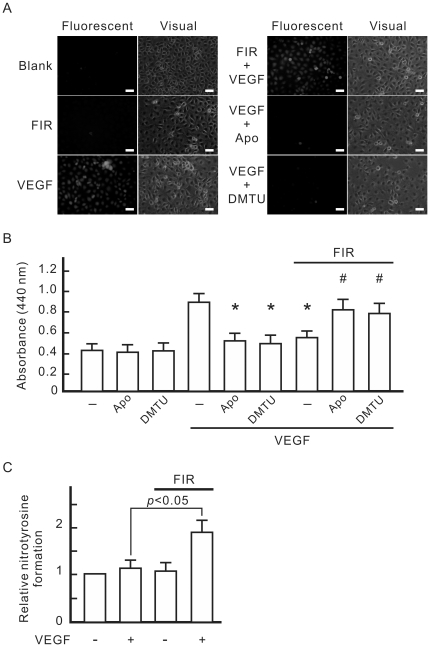
Involvement of VEGF-induced ROS in the inhibitory effect of FIR on VEGF-induced proliferation in HUVECs. (A) Detection of VEGF-induced intracellular reactive oxygen species (ROS). HUVECs were incubated for 30 min with culture medium containing DCF, and then treated with 10 ng/ml VEGF, 1 mM apocynin (Apo), 10 mM dimethylthiourea (DMTU), or FIR exposure at 0.13 mW/cm^2^ for 30 min as indicated. Cells were observed by fluoromicroscopy with excitation and emission wavelengths of 485 and 530 nm, respectively, and a photomicrograph is shown in the right panel of each group. Scale bar = 50 µm. (B) The influence of ROS scavengers on VEGF-induced cell proliferation. HUVECs were pretreated as indicated, and then cultured overnight. Data are shown as the mean±S.D. from six experiments. * *p*<0.05 vs. the group treated with VEGF alone. ^#^ *p*<0.05 vs. the group treated with VEGF and FIR exposure. (C) Detection of nitrotyrosine formation. HUVECs were pretreated as indicated, and then cultured for 1 h. The nitrotyrosine formation data obtained from ELISA measurements are expressed as relative quantity (means±S.D. of 9 determinations in three cell preparations).

Intracellular ROS detection showed that VEGF-induced ROS generation in HUVECs was significantly inhibited by an NADPH oxidase inhibitor, apocynin, and a hydroxyl radical scavenger, dimethylthiourea (DMTU), but was not influenced by FIR exposure (Fig. 4A). Both apocynin and DMTU inhibited VEGF-induced proliferation in HUVECs (Fig. 4B). In ROS scavenger-treated HUVECs with VEGF treatment, FIR exposure significantly increased cell proliferation (Fig. 4B). This result reveals that VEGF-induced ROS generation plays a role in the inhibitory effect of FIR on VEGF-induced proliferation in HUVECs. Additionally, NO reacts with superoxide anion radical to form peroxynitrite. Intracellular peroxynitrite can modify proteins by interacting with and nitrating tyrosine residues to form 3-nitrotyrosine. We also monitored nitrotyrosine formation in HUVECs treated with VEGF and FIR. As shown in Fig. 4C, nitrotyrosine formation significantly increased in HUVECs treated with VEGF and FIR together. Neither VEGF nor FIR alone influenced nitrotyrosine formation in HUVECs.

### FIR-induced eNOS phosphorylation involves PI3K-dependent Akt activation

**Figure 5:**
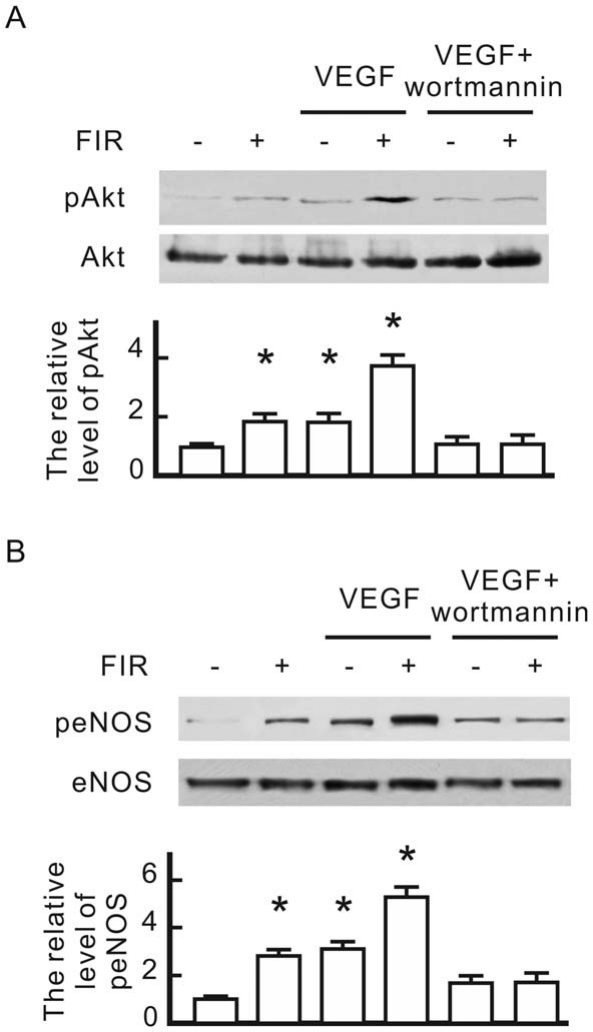
The influence of wortmannin on FIR-induced eNOS phosphorylation in HUVECs. HUVECs were pretreated with vascular endothelial growth factor (VEGF) (10 ng/ml), wortmannin (1 µM), or FIR exposure for 30 min as indicated, and then cultured for 1 h. (A) Western blotting of phospho-Akt. (B) Western blotting of phospho-eNOS. Akt and eNOS were detected as loading controls, and quantified phospho-eNOS and phospho-Akt expression relative to eNOS and Akt respectively. By comparison with the control without FIR exposure, relative levels of phospho-eNOS and phospho-Akt were obtained and are shown as the mean±S.D. from six determinations in three cell preparations. * *p*<0.05 vs. the control without FIR exposure.

To understand the molecular mechanisms underlying FIR-induced eNOS activation, we focused on PI3K-dependent activation of Akt, a known eNOS kinase. As shown in Fig. 5A, both FIR and VEGF increased Akt phosphorylation in HUVECs, and FIR further increased Akt phosphorylation in VEGF-treated cells. The PI3K/Akt pathway inhibitor, wortmannin, abolished Akt phosphorylation in VEGF-treated HUVECs with or without FIR exposure. Similar to the expression of Akt phosphorylation, wortmannin also significantly inhibited eNOS phosphorylation in VEGF-treated HUVECs with or without FIR exposure (Fig. 5B).

### FIR upregulated PI3K expression

**Figure 6:**
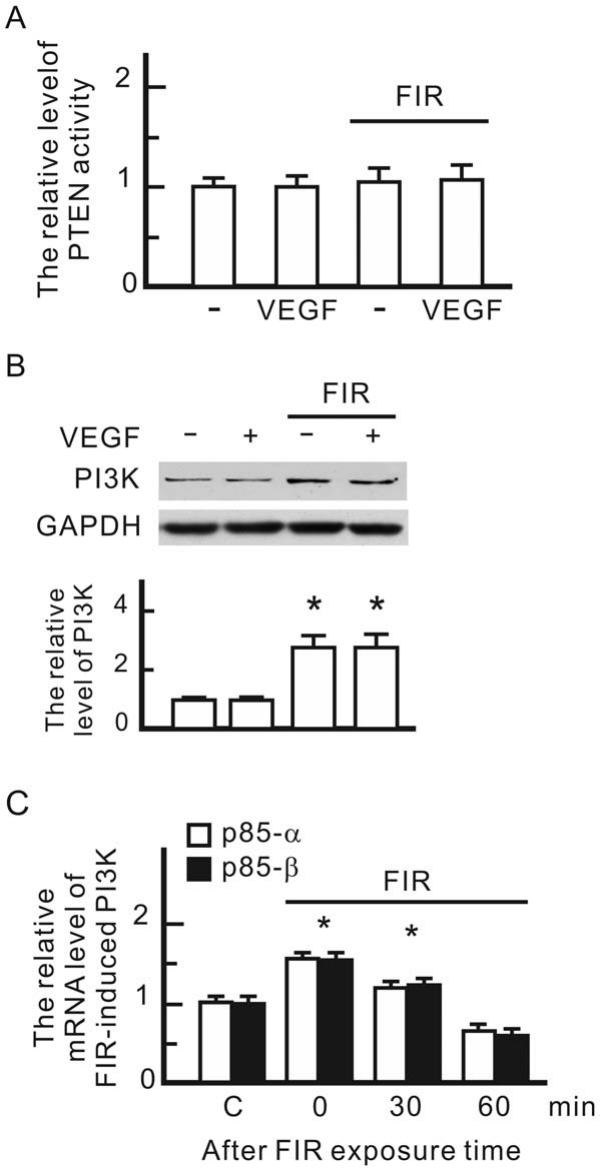
The influence of FIR on the PI3K signaling pathway in HUVECs. (A) FIR exposure-influenced phosphatase and tensin homolog (PTEN) activity. HUVECs were treated with vascular endothelial growth factor (VEGF) (10 ng/ml) or FIR exposure as indicated for 30 min. PTEN activity in each sample was detected by PTEN Malachite Green Assay Kit. The relative level of FIR-induced PTEN activity is shown as the mean±S.D. from six experiments. (B) Western blotting of PI3K p85. GAPDH was detected as a loading control. (C) FIR exposure-induced RNA levels of PI3K p85. HUVECs were pretreated with FIR exposure for 30 min, and then cultured for the indicated periods. The mRNA quantity of PI3K in each sample was detected by a qPCR with specific primers for the PI3K subunits p85α and p85β. The relative mRNA level of FIR-induced PI3K is shown as the mean±S.D. from six experiments. C, control. * *p*<0.05 vs. the control without FIR exposure.

The influence of FIR on PI3K signaling pathways was investigated by monitoring phosphatase and tensin homolog (PTEN) protein activity and PI3K expression. PTEN is a phosphatase which inhibits Akt signaling pathways. However, there was no influence of FIR on PTEN activity in HUVECs (Fig. 6A). A western blot analysis revealed that FIR but not VEGF significantly increased expression levels of PI3K p85 (Fig. 6B). The results of the qPCR showed that mRNA levels of PI3K p85-α and p85-β subunits significantly increased during FIR exposure, but gradually decreased after FIR exposure (Fig. 6C). This result reveals that FIR exposure upregulates the transcription of the PI3K gene in HUVECs.

### FIR exposure induced the nuclear translocation of PLZF in HUVECs

**Figure 7:**
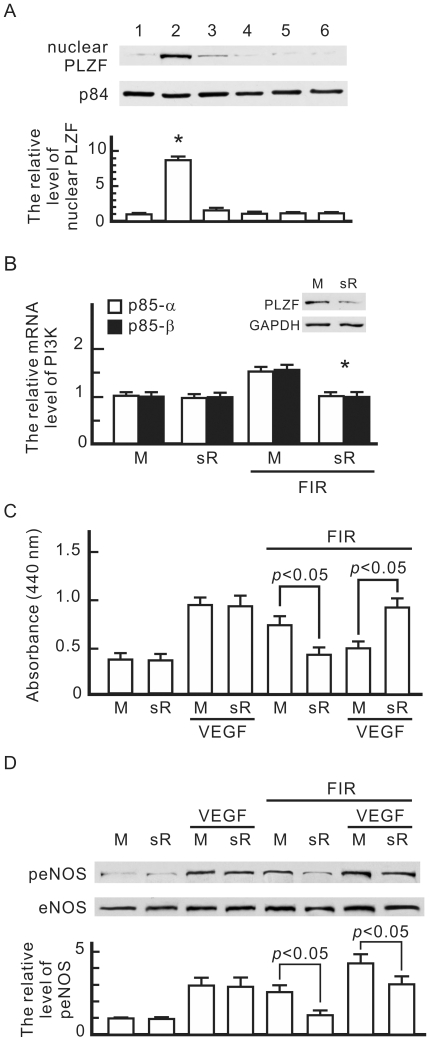
Involvement of the promyelocytic leukaemia zinc finger (PLZF) in the biological effect of FIR. (A) Western blotting of nuclear PLZF in HUVECs. HUVECs were treated as below: 1, none; 2, FIR exposure for 30 min; 3, FIR exposure for 30 min and then culture for 30 min; 4, VEGF (10 ng/ml) treatment alone for 30 min; 5, 38°C culture alone for 30 min; 6, 39°C culture alone for 30 min. Nuclear proteins were extracted and detected with a PLZF monoclonal antibody. Nuclear matrix protein p84 was detected as a loading control. By comparison with the control without FIR exposure, relative levels of nuclear PLZF were obtained and are shown as the mean±S.D. from six determinations in three cell preparations. * *p*<0.05 vs. the control without FIR exposure. (B) PLZF siRNA transfection-influenced RNA levels of PI3K p85. HUVECs were transfected with PLZF siRNA (sR) or mock control RNA (M). PLZF expression was monitored by a western blot analysis. Transfected cells were treated with or without FIR exposure as indicated for 30 min. The mRNA quantity of PI3K in each sample was detected by a qPCR with specific primers for PI3K subunits p85α and p85β. The relative mRNA level of FIR-induced PI3K is shown as the mean±S.D. from six experiments. * *p*<0.05 vs. the mock control with FIR exposure. (C) PLZF siRNA transfection-influenced cell proliferation in HUVECs. Transfected cells were pretreated with VEGF (10 ng/ml) or FIR exposure as indicated for 30 min, and then cultured overnight. Data are shown as the mean±S.D. from six experiments. (D) PLZF siRNA transfection-influenced phospho-eNOS in HUVECs. Transfected cells were treated as indicated and then cultured for 1 h. We detected eNOS as a loading control, and quantified phospho-eNOS expression relative to eNOS. By comparison with the mock control without VEGF treatment and FIR exposure, relative levels of phospho-eNOS were obtained and are shown as the mean±S.D. from six determinations in three cell preparations.

PI3K-p85 is known to be positively regulated by PLZF [18]. We further monitored the influence of FIR exposure on PLZF activation. As shown in Fig. 7A, FIR exposure dramatically induced the translocation of PLZF to nuclei in HUVECs. This translocation almost disappeared after FIR exposure for 30 min. VEGF and treatments at 38 and 39°C did not induce PLZF translocation. We used PLZF siRNA transfection to block PLZF expression in HUVECs, and then found that FIR exposure failed to elevate mRNA levels of the PI3K p85-α and p85-β subunits (Fig. 7B). PLZF siRNA transfection blocked FIR-induced proliferation and the inhibitory effect of FIR on VEGF-induced proliferation in HUVECs (Fig. 7C). PLZF siRNA transfection also reduced FIR-induced eNOS phosphorylation, but did not influence VEGF-induced eNOS phosphorylation (Fig. 7D). These results reveal that PLZF activation plays a critical role in the biological effects of FIR.

## Discussion

Based on our results, the FIR exposure at 0.13 mW/cm^2^ for 30 min achieved the maximum inhibitory effect of FIR on VEGF-induced proliferation in HUVECs. A higher FIR intensity or longer exposure time was unable to increase the inhibitory effect of FIR. FIR exposure also reduced VEGF-induced ERK1/2 phosphorylation, and increased the phosphorylation of eNOS. Applications of the NOS inhibitor and ROS scavengers showed that the inhibition effect of FIR was associated with eNOS-mediated NO and VEGF-induced ROS. Although FIR exposure usually accompanies thermal transmission, we found thermal pretreatments slightly increased VEGF-induced proliferation in HUVECs. The thermal effect of 38 or 39°C did not influence phosphorylation levels of ERK1/2 or eNOS. Compared to the influence of FIR on the eNOS signaling pathway, these results show that the biological effects of FIR in HUVECs do not result from a thermal effect. Moreover, we found that FIR exposure induced the nuclear translocation of PLZF which increased PI3K expression. This PI3K increase activated Akt which phosphorylated eNOS to generate NO in HUVECs. Pretreatment at 38 or 39°C did not induce PLZF translocation. Therefore, a non-thermal effect of FIR inhibits VEGF-induced proliferation via PLZF activation.

PLZF is a sequence-specific DNA-binding transcriptional factor. It comprises 673 amino acids and contains nine Kruppel-like C_2_H_2_ zinc finger domains and a POZ/BTB protein-binding domain [19], [20]. PLZF was identified as a chromosomal fusion partner with retinoic acid receptor (RAR)α in acute promyelocytic leukemia, a disease marked by an accumulation of undifferentiated myeloid blasts [20], [21]. Although RARα activates key genes required for normal myelopoiesis, the PLZF-RARα fusion protein represses the expression of some of these genes in a dominant negative manner. Senbonmatsu et al. reported that angiotensin II stimulation provokes internalization of angiotensin II type 2 receptor and PLZF, and translocation of PLZF to the nucleus [18]. Nuclear PLZF activates the PI3K p85-α gene leading to subsequent activation of protein synthesis. Schefe et al. demonstrated a novel renin/prorenin receptor (RR) signal transduction pathway involving direct protein-protein interactions between the RR and PLZF, and the nuclear translocation of PLZF upon renin stimulation [22]. Downstream effects of RR activation by renin include repression of the RR itself and induction of the PI3K p85-α subunit. In the present study, we also found that the nuclear translocation of PLZF induced expressions of the PI3K p85-α and p85-β subunits at the transcriptional level. However, no study has revealed the connection between FIR exposure and PLZF. Our findings provide the first evidence that FIR exposure induces the nuclear translocation of PLZF in HUVECs. FIR exposure may exert multiple biological functions in HUVECs via the nuclear translocation of PLZF.

NO is a free radical species that diffuses and concentrates in the hydrophobic core of low-density lipoproteins to serve as a potent antioxidant [23]. Endothelium-derived NO is a paracrine factor that controls vascular tone, inhibits platelet function, prevents leukocyte adhesion, and reduces intimal proliferation [24]. Endothelial NOS-mediated NO generation also plays a crucial role in the process of VEGF-induced angiogenesis [25]. Our results showed that the NOS inhibitor, L-NAME, inhibited both VEGF-induced NO generation and proliferation in HUVECs. FIR-induced NO generation also increased HUVEC proliferation without VEGF treatment (Fig. 2). However, a higher concentration of NO induced by FIR and VEGF together did not induce greater HUVEC proliferation, but showed an inhibitory effect instead. The influence of NO on HUVEC proliferation may be concentration-dependent. Additionally, VEGF-induced ROS plays a role in inhibiting HUVEC proliferation while treating with FIR and VEGF together (Fig. 4B). In principle, a high concentration of NO can react with VEGF-induced ROS to generate peroxynitrite production in HUVECs. Peroxynitrite, a product of the diffusion-limited reaction between NO and superoxide anions, mediates oxidative modifications in lipid systems including cell membranes and lipoproteins [26]. Intracellular peroxynitrite can modify proteins by interacting with and nitrating tyrosine residues to form 3-nitrotyrosine. Our results showed that the combination of FIR and VEGF treatments significantly induced nitrotyrosine formation in HUVECs (Fig. 4C). Peroxynitrite exhibits a wide array of tissue-damaging effects ranging from lipid peroxidation, inactivation of enzymes and ion channels via protein oxidation and nitration to inhibition of mitochondrial respiration [27]. Agbani et al. found that >2 µM peroxynitrite caused apoptotic cell death independent of p38 MAP kinase activation in pulmonary artery cells [28]. Therefore, the mechanisms of lipid oxidation and nitration by peroxynitrite induced by FIR and VEGF together may interfere with HUVEC proliferation.

FIR therapy was found to improve inadequate access flow and survival of an AVF in HD patients, although the mechanism of its non-thermal effects is still unknown [6]. Malfunction of vascular access results primarily from venous stenosis, which is caused by intimal hyperplasia with subsequent impairment of blood flow and a thrombosis [7]. It is possible that the NO-mediated inhibitory effect of FIR on VEGF-induced proliferation in vascular endothelial cells can reduce VEGF-mediated intimal hyperplasia with hemodialysis graft placement. Additionally, NO induces vasodilation, inhibits platelet aggregation, prevents neutrophil/platelet adhesion to endothelial cells, inhibits smooth muscle cell proliferation and migration, regulates programmed cell death, and helps maintain the endothelial cell barrier function [29]. Thus, NO bioavailability is crucial for maintaining vascular endothelial health and function [30]. In theory, FIR therapy can reduce intimal hyperplasia, prevent venous stenosis, and increase access flow of the AVF in HD patients via PI3K/Akt-activated NO generation, and is also a potential remedy for other vascular disorders such as peripheral obstructive arterial disease.

Because thermal transmission always accompanies FIR emission, FIR therapy often possesses a thermal effect. In this study, we evaluated the thermal effect at temperatures (37∼39°C) generated by FIR exposure. Unlike the biological effect of FIR, the thermal effect slightly increased VEGF-induced proliferation in HUVECs. The influence of the thermal effect differed from the biological effect of FIR on PLZF translocation and PI3K/Akt signaling pathways. Actually, the increase in FIR intensity or exposure time gradually reduced the inhibitory effect of FIR. This may have resulted from the thermal effect caused by FIR exposure because the increase in FIR intensity or exposure time significantly raised the temperature of the culture medium. Thus, we suggest that the temperature increase should be limited in FIR therapy on AVFs in HD patients to achieve maximum therapeutic effects.

In conclusion, the present study reveals that FIR exposure induced the nuclear translocation of PLZF which upregulated PI3K to activate Akt, and then activated eNOS to induce NO generation. This NO generation combined with VEGF-induced ROS generation resulted in inhibition of VEGF-induced proliferation in HUVECs. Through a PLZF-mediated pathway, FIR therapy is a potential therapeutic modality to maintain vascular endothelial health and function.

## References

[1] Imamura M, Biro S, Kihara T, Yoshifuku S, Takasaki K (2001). Repeated thermal therapy improves impaired vascular endothelial function in patients with coronary risk factors.. J Am Coll Cardiol.

[2] Ikeda Y, Biro S, Kamogawa Y, Yoshifuku S, Eto H (2005). Repeated sauna therapy increases arterial endothelial nitric oxide synthase expression and nitric oxide production in cardiomyopathic hamsters.. Circ J.

[3] Kihara T, Biro S, Ikeda Y, Fukudome T, Shinsato T (2004). Effects of repeated sauna treatment on ventricular arrhythmias in patients with chronic heart failure.. Circ J.

[4] Akasaki Y, Miyata M, Eto H, Shirasawa T, Hamada N (2006). Repeated thermal therapy up-regulates endothelial nitric oxide synthase and augments angiogenesis in a mouse model of hindlimb ischemia.. Circ J.

[5] Yu SY, Chiu JH, Yang SD, Hsu YC, Lui WY (2006). Biological effect of far-infrared therapy on increasing skin microcirculation in rats.. Photodermatol Photoimmunol Photomed.

[6] Lin CC, Chang CF, Lai MY, Chen TW, Lee PC (2007). Far-infrared therapy: a novel treatment to improve access blood flow and unassisted patency of arteriovenous fistula in hemodialysis patients.. J Am Soc Nephrol.

[7] Roy-Chaudhury P, Sukhatme VP, Cheung AK (2006). Hemodialysis vascular access dysfunction: a cellular and molecular viewpoint.. J Am Soc Nephrol.

[8] Rekhter M, Nicholls S, Ferguson M, Gordon D (1993). Cell proliferation in human arteriovenous fistulas used for hemodialysis.. Arterioscler Thromb.

[9] Wang Y, Krishnamoorthy M, Banerjee R, Zhang J, Rudich S (2008). Venous stenosis in a pig arteriovenous fistula model–natomy, mechanisms and cellular phenotypes.. Nephrol Dial Transplant.

[10] Schwartz SM, deBlois D, O'Brien ER (1995). The intima. Soil for atherosclerosis and restenosis.. Circ Res.

[11] Sukhatme VP (1996). Vascular access stenosis: prospects for prevention and therapy.. Kidney Int.

[12] Couper LL, Bryant SR, Eldrup-Jorgensen J, Bredenberg CE, Lindner V (1997). Vascular endothelial growth factor increases the mitogenic response to fibroblast growth factor-2 in vascular smooth muscle cells in vivo via expression of fms-like tyrosine kinase-1.. Circ Res.

[13] Lindner V, Reidy MA (1991). Proliferation of smooth muscle cells after vascular injury is inhibited by an antibody against basic fibroblast growth factor.. Proc Natl Acad Sci U S A.

[14] Guarda E, Katwa LC, Campbell SE, Tanner MA, Webel RM (1996). Extracellular matrix collagen synthesis and degradation following coronary balloon angioplasty.. J Mol Cell Cardiol.

[15] Jawien A, Bowen-Pope DF, Lindner V, Schwartz SM, Clowes AW (1992). Platelet-derived growth factor promotes smooth muscle migration and intimal thickening in a rat model of balloon angioplasty.. J Clin Invest.

[16] Misra S, Fu AA, Puggioni A, Karimi KM, Mandrekar JN (2008). Increased shear stress with upregulation of VEGF-A and its receptors and MMP-2, MMP-9, and TIMP-1 in venous stenosis of hemodialysis grafts.. Am J Physiol Heart Circ Physiol.

[17] Cheng TH, Leung YM, Cheung CW, Chen CH, Chen YL (2009). Propofol Depresses Angiotensin II-induced Cell Proliferation in Rat Cardiac Fibroblasts.. Anesthesiology.

[18] Senbonmatsu T, Saito T, Landon EJ, Watanabe O, Price E (2003). A novel angiotensin II type 2 receptor signaling pathway: possible role in cardiac hypertrophy.. EMBO J.

[19] Chen Z, Brand NJ, Chen A, Chen SJ, Tong JH (1993). Fusion between a novel Kruppel-like zinc finger gene and the retinoic acid receptor-alpha locus due to a variant t(11;17) translocation associated with acute promyelocytic leukaemia.. EMBO J.

[20] Melnick A, Licht JD (1999). Deconstructing a disease: RARalpha, its fusion partners, and their roles in the pathogenesis of acute promyelocytic leukemia.. Blood.

[21] Costoya JA, Pandolfi PP (2001). The role of promyelocytic leukemia zinc finger and promyelocytic leukemia in leukemogenesis and development.. Curr Opin Hematol.

[22] Schefe JH, Menk M, Reinemund J, Effertz K, Hobbs RM (2006). A novel signal transduction cascade involving direct physical interaction of the renin/prorenin receptor with the transcription factor promyelocytic zinc finger protein.. Circ Res.

[23] Rubbo H, O'Donnell V (2005). Nitric oxide, peroxynitrite and lipoxygenase in atherogenesis: mechanistic insights.. Toxicology.

[24] Forstermann U (2010). Nitric oxide and oxidative stress in vascular disease.. Pflugers Arch.

[25] Bouloumie A, Schini-Kerth VB, Busse R (1999). Vascular endothelial growth factor up-regulates nitric oxide synthase expression in endothelial cells.. Cardiovasc Res.

[26] Rubbo H, Trostchansky A, O'Donnell VB (2009). Peroxynitrite-mediated lipid oxidation and nitration: mechanisms and consequences.. Arch Biochem Biophys.

[27] Virag L, Szabo E, Gergely P, Szabo C (2003). Peroxynitrite-induced cytotoxicity: mechanism and opportunities for intervention.. Toxicol Lett.

[28] Agbani E, Coats P, Wadsworth RM (2011). Threshold of peroxynitrite cytotoxicity in bovine pulmonary artery endothelial and smooth muscle cells.. Toxicol In Vitro.

[29] Rosselli M, Keller PJ, Dubey RK (1998). Role of nitric oxide in the biology, physiology and pathophysiology of reproduction.. Hum Reprod Update.

[30] Rush JW, Denniss SG, Graham DA (2005). Vascular nitric oxide and oxidative stress: determinants of endothelial adaptations to cardiovascular disease and to physical activity.. Can J Appl Physiol.

